# Melatonin Improves Drought Stress Tolerance of Tomato by Modulating Plant Growth, Root Architecture, Photosynthesis, and Antioxidant Defense System

**DOI:** 10.3390/antiox11020309

**Published:** 2022-02-03

**Authors:** Muhammad Ahsan Altaf, Rabia Shahid, Ming-Xun Ren, Safina Naz, Muhammad Mohsin Altaf, Latif Ullah Khan, Rahul Kumar Tiwari, Milan Kumar Lal, Muhammad Adnan Shahid, Ravinder Kumar, Muhammad Azher Nawaz, Mohammad Shah Jahan, Basit Latief Jan, Parvaiz Ahmad

**Affiliations:** 1 School of Horticulture, Hainan University, Haikou 570228, China; ahsanaltaf8812@gmail.com; 2 School of Management, Hainan University, Haikou 570228, China; rabiashahidlhr@yahoo.com; 3 Center for Terrestrial Biodiversity of the South China Sea, Hainan University, Haikou 570228, China; 4 College of Ecology and Environment, Hainan University, Haikou 570228, China; mohsinaltaf641@gmail.com; 5 Department of Horticulture, Bahauddin Zakariya University, Multan 60800, Pakistan; Safina_bzu@yahoo.com; 6 College of Tropical Crop, Hainan University, Haikou 570228, China; latif.hainu3103@gmail.com; 7 ICAR-Indian Agricultural Research Institute, New Delhi 110012, India; rahultiwari226@gmail.com (R.K.T.); milan2925@gmail.com (M.K.L.); chauhanravinder97@gmail.com (R.K.); 8 ICAR-Central Potato Research Institute, Shimla 171001, India; 9 Department of Agriculture, University of New Hampshire, Durham, NC 03824, USA; muhammad.shahid@unh.edu; 10 Department of Horticulture, College of Agriculture, University of Sargodha, Sargodha 171001, Pakistan; azher490@gmail.com; 11 Department of Horticulture, Faculty of Agriculture, Sher-e-Bangla Agricultural University, Dhaka 1207, Bangladesh; shahjahansau@gmail.com; 12 Department of Clinical Pharmacy, College of Pharmacy, King Saud University, Riyadh 11451, Saudi Arabia; Bjan@ksu.edu.sa; 13 Botany and Microbiology Department, King Saud University, Riyadh 11451, Saudi Arabia; parvaizbot@yahoo.com

**Keywords:** tomato, photosynthesis, root growth, oxidative damage, melatonin, drought, gene expression

## Abstract

Tomato is an important vegetable that is highly sensitive to drought (DR) stress which impairs the development of tomato seedlings. Recently, melatonin (ME) has emerged as a nontoxic, regulatory biomolecule that regulates plant growth and enhances the DR tolerance mechanism in plants. The present study was conducted to examine the defensive role of ME in photosynthesis, root architecture, and the antioxidant enzymes’ activities of tomato seedlings subjected to DR stress. Our results indicated that DR stress strongly suppressed growth and biomass production, inhibited photosynthesis, negatively affected root morphology, and reduced photosynthetic pigments in tomato seedlings. Per contra, soluble sugars, proline, and ROS (reactive oxygen species) were suggested to be improved in seedlings under DR stress. Conversely, ME (100 µM) pretreatment improved the detrimental-effect of DR by restoring chlorophyll content, root architecture, gas exchange parameters and plant growth attributes compared with DR-group only. Moreover, ME supplementation also mitigated the antioxidant enzymes [APX (ascorbate peroxidase), CAT (catalase), DHAR (dehydroascorbate reductase), GST (glutathione S-transferase), GR (glutathione reductase), MDHAR (monodehydroascorbate reductase), POD (peroxidase), and SOD (superoxide dismutase)], non-enzymatic antioxidant [AsA (ascorbate), DHA (dehydroascorbic acid), GSH (glutathione), and GSSG, (oxidized glutathione)] activities, reduced oxidative damage [EL (electrolyte leakage), H_2_O_2_ (hydrogen peroxide), MDA (malondialdehyde), and O_2_^•−^ (superoxide ion)] and osmoregulation (soluble sugars and proline) of tomato seedlings, by regulating gene expression for *SOD*, *CAT*, *APX*, *GR*, *POD*, *GST*, *DHAR*, and *MDHAR*. These findings determine that ME pretreatment could efficiently improve the seedlings growth, root characteristics, leaf photosynthesis and antioxidant machinery under DR stress and thereby increasing the seedlings’ adaptability to DR stress.

## 1. Introduction

In recent decades, water has been considered a vital environmental factor, and its deficiency is known as drought stress (DR) which leads to restricted plant growth, yield, and quality, mainly due to various morphological, physiological, anatomical, and biochemical responses. Compared with other environmental stresses, DR stress is particularly prominent in abiotic stress [1]. Water scarcity adversely influences plant growth by reducing relative water content (RWC), root length, seedling stem, leaf area, and leaf water potential [2]. Plants exhibit multiple and interconnected responses towards DR stress. DR stress can be fatal for underdeveloped root systems of seedlings in the spring season [3]. Earlier studies revealed that DR stress can cause reduced pigment content, imbalanced ion homeostasis, decreased transpiration, stomatal closure, cell enlargement reduction, reduce canopy size, and ultimately lead towards plant death [4]. The impact of DR stress varies with the intensity and the growth stage of plants [5].

The excessive accumulation of ROS in plants under water-deficient conditions can cause oxidative damage which might lead to significant damage to the cellular organelles [6]. The ROS molecules include H_2_O_2_, O_2_^•−^, and other oxygen-containing molecules which interact with the membrane system of the and damage mainly the macromolecules present in the cell [7]. Excessive accumulation ROS cause oxidative damage to the electron transport chain, enhance lipid peroxidation in chloroplast and mitochondria, inactivates enzyme activity, proteins, and nucleic acids, and ultimately decreases photosynthesis and yield of crops [8]. The antioxidant enzymes (APX, CAT, DHAR, GR, GST, MDHAR, POD, and SOD) and non-enzymatic antioxidants [AsA, DHA, GSH, and GSSG] molecules effectively reduce the ROS accumulation, thereby balancing the ROS synthesis and signaling to provide enhance DR-stress tolerance in plants [9]. Additionally, the accumulation of osmolyte also helps effectively against DR stress to prevent oxidative meditative damage in the plant. Soluble sugars, proline, and proteins are the most common osmolytes which interact at the cellular level by decreasing membrane permeability under mild water scarcity, thus maintaining water balances of crops under DR stress [10]. 

Recent trends of climate change have significantly affected crop yield and among these various abiotic conditions, DR stress is one of the main culprits [11]. To circumvent this condition, various agronomic strategies are needed for improving drought tolerance in plants which include employing phytohormones or bio-stimulators in crop production. Such phytohormones pose a positive impact on growth regulation and resilience development in plants by boosting various physiological, biochemical and molecular processes [12]. Thus, for better tolerance towards DR stress, it is of great significance to explore the effect and mechanism of these phytohormones.

Melatonin is a natural, multifunctional, nontoxic, regulatory, and universal biomolecule, having low molecular weight with pleiotropic effects in the plant kingdom [13]. Hitherto, ME is evidenced to enhance the resistance to different abiotic stresses such as DR, heat, cold, salt, heavy metals, chemicals, and pathogens [14,15,16]. Furthermore, ME promoted root morphology seed germination, photosynthesis, seedlings growth, delay in leaf senescence, antioxidant efficiency, fruit maturation and rhizogenesis at cellular and tissue level [17,18,19]. Specifically, the increased ROS homeostasis and enhanced antioxidant capacity were related to higher ME content in DR-affected seedlings, such as *Actinidia deliciosa* [20], *Althaea rosea* [21] *Eriobotrya japonica* [22], and *Carthamus tinctorius* [23].

Previous studies revealed that under drought stress, ME enhanced plant growth attributes in tomato [24], improved the root architecture system in kiwifruit [25], protected mineral balance in *C. cathayensis* [26], increased photosynthetic efficiency in *E. japonica* [22], reduced lipid peroxidation and ROS accumulation in *Moringa oleifera* [27], promoted antioxidant enzymes system in fenugreek [7], increased glutathione, and ascorbic acid content in kiwifruit seedlings [20], enhanced osmoregulatory substances in tomato [28], protected grana lamella of chloroplast in wheat [29], preserved the chloroplast structure in *Brassica napus* [30], and significantly regulated the antioxidant enzymes (APX, CAT, DHAR, GST, GR, MDHAR, POD, SOD) and non-enzymatic antioxidant (AsA, GSH) genes in *Solanum lycopersicum* and *Carya cathayensis* [19,26].

In the solanaceous family, after potato, tomato is the 2nd most important cultivated and extensively used vegetable around the world. Like other crops, water scarcity prominently causes an inhibitory effect on the physiological, morphological, biochemical, and anatomical structure of tomato [31]. Tomato is highly sensitive to water scarcity, particularly at the germination and seedling growth and seed germination stage [32]. However, information about how ME regulates the physiological, morphological, biochemical, and anatomical changes in tomato under water deficit conditions remains elusive. Therefore, in this study, we aimed to explore the impacts of ME in enhancing DR tolerance and analyze the mechanism of increased DR tolerance induced by ME. Our main focus was to investigate the photosynthetic performance, membrane damage of tomato seedlings, root architecture system, the chloroplast structure, and ROS homeostasis regulated by ME under DR stress. Precisely, we also examined enzymes related to the Haliwell Asada pathway and their genes expression. The present study has attempted to contribute to the elucidation of the biological function of ME in building resilience against DR stress.

## 2. Materials and Methods

### 2.1. Plant Material

In the present study, the tomato cultivar “Fenli” was collected from Minghao seed store, Hainan, China. The germinated seeds were sown in vermiculite-filled trays with a 50-cell plug in a controlled growth chamber. After the development of the second-true-leaf, equal size tomato seedlings were transferred into the vermiculite-filled black plastic pots (with height = 8.5 cm, top and bottom diameter = 10 and 7 cm, respectively), having one emerged seedling/pot. Subsequently, all the pots were placed in a greenhouse with specific conditions (25 °C ± 5 temperature; 16/8 h dark/light period; and 55–90% relative humidity). The pre-cultivation period was 10 days, with the aim of allowing seedlings get adapted to new conditions, with watering (80 mL per plant) at two days’ interval using Hoagland’s nutrient solution (pH 5.8 ± 0.1).

### 2.2. Experimental Design

The treatment plan was implemented after ten days, when the seedlings were attained at the four-true-leaf stage. The treatments are followed in the current study including: (1) CK (control) treatment included the seedlings with full water application during the complete span of the experiment, (2) DR (drought) treatment included the plants which were first given water fully for eight days, followed by up to two weeks’ water-withholding, (3) ME + DR (melatonin + drought) was the treatment in which ME pretreatment was given to seedlings with 100 μM solution of ME (80 mL per plant) [33]. This pretreatment continued four times, with a 2-days’ interval, followed by up to two weeks’ water-withholding. Each treatment comprised of 3 replicates, with 8 plants per replicate. Further, 0 day was the day when irrigation-withholding was started. After two-weeks of DR treatment, collection of leaf samples was done to perform different analysis, including both biochemical and physiological tests. 

### 2.3. Vegetative Growth

After two weeks of DR-stress, seedlings were washed with tap water. Roots and shoots were separated for measuring both fresh and dry weight. After measuring fresh weight, root and shoot were immediately placed in an individual paper bag and put in an oven for drying. The dry weight of root and shoot were recorded after drying of samples at 105 °C for 15 min, and 75 °C for three days [34]. Plant height was estimated using a measuring tape.

### 2.4. Leaf Gas Exchange and Pigments

Photosynthetic parameters including Pn (net photosynthetic rate), Ci (intercellular CO_2_ concentration), Gs (stomatal conductance) and Tr (transpiration rate) were assayed (9 to 11 a.m.) by operating a portable photosynthesis system, namely CIRAS-3, bought from Hansatech Co., Amesbury, MA, USA, with controlled measuring chamber conditions (25 °C leaf temperature, 360 μM/mol CO_2_ concentration, and 800 μM/m^2^/s photosynthetic photon-flux density).

The method of Arnon [35] was followed to measure chlorophyll and carotenoid content. Concisely, slicing of leaf samples (0.1 g) was done, and sliced samples were stored in a dark place after placement in glass test tubes and addition of acetone (80%) per tube. The samples were stored in the dark until they were completely discolored, followed by the centrifugation of sample extract. Finally, Lambda 25 UV/VIS Spectrophotometer was employed to calculate chlorophyll a (Chl a), Chlorophyll b (Chl b), and carotenoid (Caro) via measuring absorbance at 645, 663, and 440 nm, respectively.

### 2.5. Root Morphology and Root Activity

After two-weeks of DR-stress, roots of three plants were taken and washed using tap water, to study root morphological traits. Scanning of roots was done using a root scanner, followed by evaluating the scanned images using a root image analysis software (Epson Expression 11000XL, Regent Instruments, Québec, QC, Canada), to obtain the root morphological parameters [36].

The TTC (triphenyl tetrazolium chloride) method, as explained by Comas et al. [37], was followed to estimate root activity (capacity of root deoxidization measured in mg g^−1^ h^−1^ FW). Briefly, root samples (500 mg) were dipped in 10 mL of a mixed solution of phosphate buffer and TTC (0.4%), which is mixed in a 1:1 ratio. After that, the sample was placed in the dark for one to three hours (37 °C), followed by the addition of 2 mL of H_2_SO_4_ (1 mol L^−1^) to avoid the reaction of the solution. Finally, dipped roots were blended and transferred into 10 mL of ethyl acetate cleaning solution in a tube, and the spectrophotometer was used to record solution’ absorption at 485 nm.

### 2.6. Leaf Relative Water Content and Soluble Sugar

According to the method of Barrs and Weatherley [38], the following formula was used to calculate leaves’ *RWC* (relative water content) (Equation (1)):

(1) $$RWC\;\left(\%\right)=\left[\frac{FW-DW}{SW-DW}\right]\times 100$$

where FW = fresh weight; DW = dry weight; SW = saturated weight in water.

The anthrone method of Shi et al. [39] was employed to perform soluble sugar content. In brief, 80% (*V*/*V*) ethanol (2 mL) was added to 0.1 g sample at 80 °C, and kept for 30 min. Then, anthrone (2 mL) was mixed to extract (100 µL), followed by boiling for 10 min. Finally, after recording the absorption (at 630 nm), the calibration curve of the sucrose standard was used to calculate the soluble sugar content.

### 2.7. Oxidative Stress Markers, and Proline

For calculating H_2_O_2_, O_2_^•−^, MDA, and proline contents, liquid nitrogen was used to crush frozen sample (0.1 g) into powder form, followed by isolation using 100 mM phosphate buffer (900 μL, pH 7.4) and guidelines labelled in kits (Nanjing Jiancheng Bioengineering Institute, located in Nanjing, China) of H_2_O_2_ (A064), O_2_^•−^ (A052), MDA (A003-3), and proline (A107-1), at 405, 550, 530, and 520 nm, respectively. Leaves’ EL was measured by following the method of Zhang et al. [40]. 

### 2.8. Antioxidant Enzymes Activity

To examine the antioxidant enzyme activities, powdered leaf sample (0.5 g) was homogenized using 100 mM phosphate buffer (900 μL, pH 7.4) by following kits’ description. After that, centrifugation (at 4 °C temperature, 12,000× *g* revolutions for 15 min) of homogenized samples was done, and to analyze enzymatic activities, the supernatant was shifted to new falcon tube. The guidelines mentioned in kits (A001-1, A007-1, A123-1, A062-1, A004, A084-3-1, BC0660, and BC0650) were followed to note the activities of SOD, CAT, APX, GR, GST, POD, DHAR, and MDHAR enzymes, at 550, 405, 290, 340, 412, 420, 412, and 340 nm wavelengths, respectively.

The method of Logan et al. [41] was followed to assess the levels of ascorbate (AsA) and oxidized ascorbate (DHA). The procedures of Griffith [42] were followed to measure reduced glutathione (GSH) and oxidized glutathione (GSSG) content.

### 2.9. Quantitative Real-Time PCR

Following the manufacturer’s instructions, Trizole reagent was used to extract total RNA from individual treatment leaf samples. With the help of agarose gel electrophoresis, a NanoPhotometer^®^ spectrophotometer (Implen, Westlake Village, CA, USA), the extracted RNA’ purity and quality were examined. The manufacturer protocol of Vazyme HiScript II Q RT SuperMix for qPCR (+gDNA wiper) 1st strand cDNA synthesis kit (Vazyme, Nanjing, China) was followed to reverse-transcribe the extracted RNA, for complementary DNA (cDNA) synthesis. For qRT-PCR (quantitative real-time PCR) analysis, cDNA was used as templates. The Roche FastStart Essential DNA Green Master kit (Roche, Pleasanton, CA, USA) was employed in an Mx3000 P qPCR system (Agilent Technologies, Santa Clara, CA, USA), and 96-well plates were used to perform qRT-PCR. Supplementary Table S1 provides the detail of primers followed in this study Jahan et al. [43], and Actin was used as a reference gene. The formula of Livak and Schmittgen [44], i.e., 2^−ΔΔCt^ was used to calculate the changes in relative gene expression. Three biological replications were performed for each treatment, and three technical replicates were carried out for each biological replicate. 

### 2.10. Statistical Analysis 

The statistical package SPSS version 22.0 (IBM Corporation, Armonk, NY, USA) was used for the statistical analysis of data. One-way analysis of variance (ANOVA) was completed, and the treatment means were compared using the LSD (least significant difference) test (at *p* ≤ 0.05). 

## 3. Results

### 3.1. Effect of DR Stress on Tomato Seedlings Growth and Development

**Figure 1:**
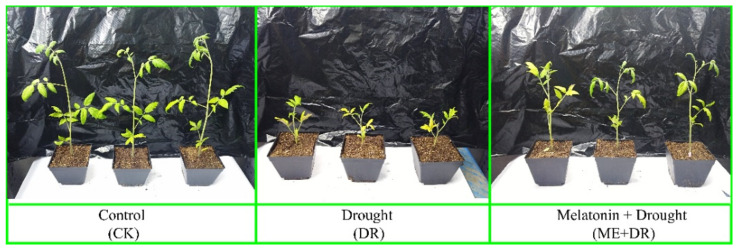
Tomato seedlings visual demonstration under ME and DR stress. Photographs of the tomato seedlings were taken.

**Figure 2:**
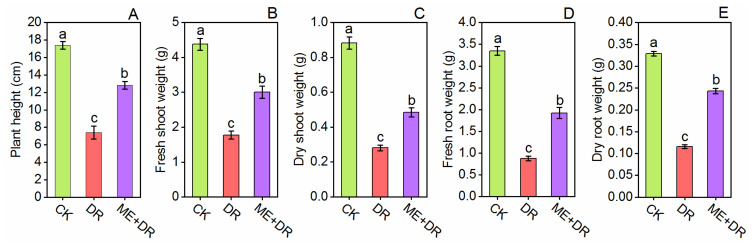
Exogenous supplementation of melatonin promoted the growth [plant height (**A**), fresh shoot weight (**B**), dry shoot weight (**C**), fresh root weight (**D**), and dry root weight (**E**)] of tomato seedlings under drought stress conditions. Means ± standard error, *n* = 3, significant differences are exhibited by lowercase letters (*p* ≤ 0.05), according to LSD test.

Drought treatment showed a significant decline in the growth of tomato seedlings compared with CK treatment (Figure 1). The tomato seedlings, when subjected to DR stress, significantly reduced in plant height (57.52%), fresh shoot weight (FSW; 61.11%), dry shoot weight (DSW; 63.58%), fresh root weight (FRW; 63.96%), and dry root weight (DRW; 64.74%), compared with CK (well-watered) plants (Figure 2) This decline in the growth of tomato seedlings was alleviated by the exogenous application of melatonin (Figure 1 and Figure 2). After pretreatment with ME, growth limitations caused by DR stress were improved, and less reductions in plant height (26.43%), FSW (30.25%), DSW (37.06%), FRW (35.63%), and DRW (26.13%) was observed (Figure 2).

### 3.2. Effect of DR Stress on Photosynthesis and Related Parameters

**Figure 3:**
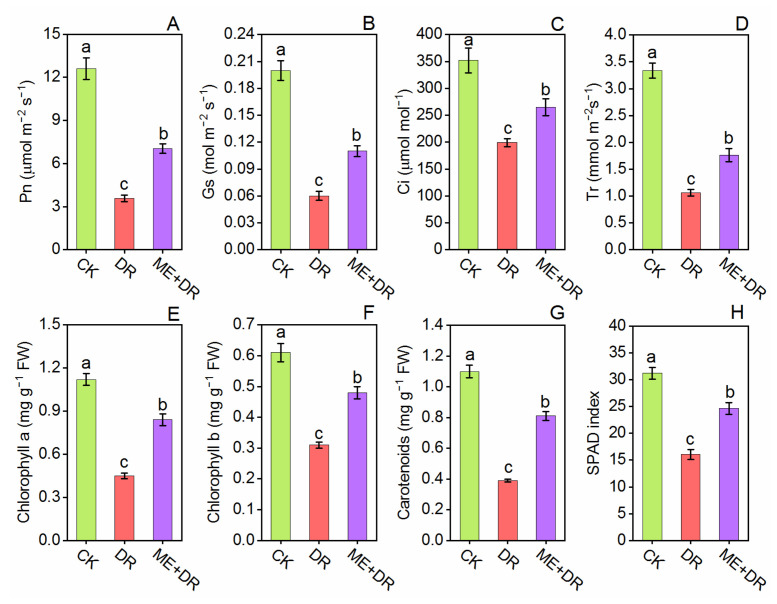
Exogenous supplementation of melatonin promoted leaf gas exchange [Pn (**A**), Gs (**B**), Ci (**C**) and Tr (**D**)] and level of pigment [chlorophyll a (**E**), chlorophyll b (**F**), carotenoids (**G**), and SPAD index (**H**)] of tomato leaves under drought stress conditions. Means ± standard error, *n* = 3, significant differences are exhibited by lowercase letters (*p* ≤ 0.05), according to LSD test.

Under DR stress, Pn, Ci, Gs, and Tr were decreased by 71.58%, 43.42%, 66.89%, and 68.04%, respectively, compared with CK seedlings (Figure 3). Conversely, when seedlings were treated with ME, the reductions of these leaf gas exchange parameters were only 44.27%, 24.59%, 43.21%, and 46.93%, respectively, as compared to the CK plants.

A similar trend of reduction in the pigment system was observed under the DR stress condition. Chl a, Chl b, and Carotenoid content in the leaves of tomatoes were reported to be sharply reduced by 59.94%, 48.63%, and 64.74%, respectively, under DR stress (Figure 3E–G). Per contra, ME-treated tomato seedlings when subjected to DR treatment, these pigments’ content significantly increased—by 86.66%, 194.68%, and 110.34%, respectively—when compared with only DR-stressed seedlings (Figure 3E–G). In DR-stressed plants, the SPAD index showed a noticeable reduction. In contrast, the ME supplementation elevated SPAD index under DR-stress (Figure 3H).

### 3.3. Changes in Root Morphology under DR Stress

**Figure 4:**
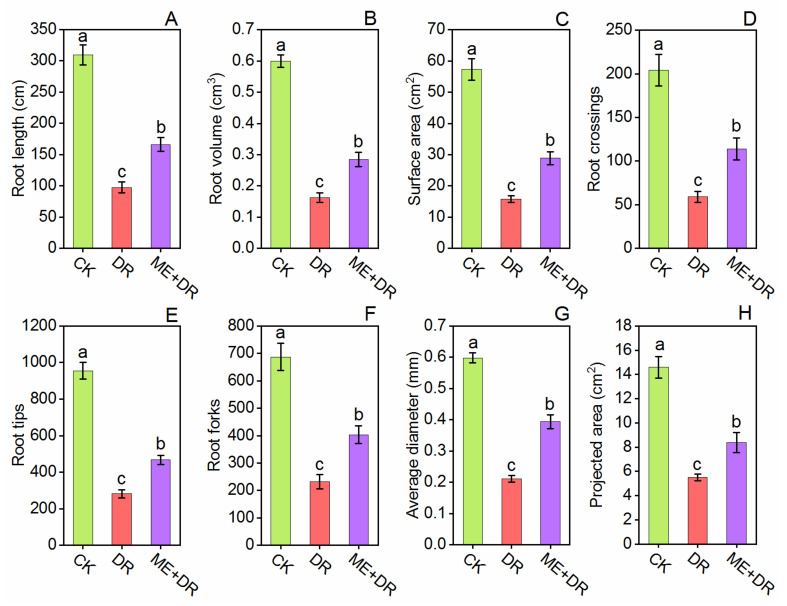
Exogenous supplementation of melatonin promoted root morphology [Root length (**A**), root volume (**B**), surface area (**C**), root crossings (**D**), root tips (**E**), root forks (**F**), average diameter (**G**), and projected area (**H**)] of tomato seedlings under drought stress conditions. Means ± standard error, *n* = 3, significant difference are exhibited by lowercase letters (*p* ≤ 0.05), according to LSD test.

The present study showed that DR treatment remarkably diminished the root morphological parameters, including root length (68.46%), root volume (72.95%), root surface area (72.40%), root crossings (71.02%), root tips (70.47%), root forks (66.22%), average root diameter (64.61%), and projected area (62.31%) compared with CK tomato seedlings (Figure 4). Interestingly, compared with DR stressed plants, these root characteristics were improved by 70.40-, 75.30-, 82.98-, 92.89-, 65.60-, 73.70-, 86.02-, and 52.36%, respectively, in ME pretreated tomato plants subjected to DR stress (Figure 4).

### 3.4. Relative Water Content, Proline, Soluble Sugars, and Root Activity Alternation Due to DR Stress

**Figure 5:**
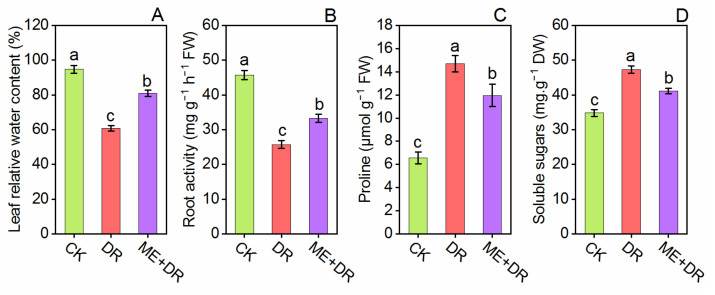
Exogenous supplementation of melatonin promoted level of relative water content (**A**), and root activity (**B**); reduced level of proline (**C**) and soluble sugar (**D**) content of tomato seedling under drought stress conditions. Means ± standard error, *n* = 3, significant difference are exhibited by lowercase letters (*p* ≤ 0.05), according to LSD test.

After two weeks of DR stress, the RWC of DR-stressed plants was noticeably reduced by 36.10%. Conversely, ME-treatment significantly improved the RWC by 34.12% compared with DR treatment (Figure 5A). As depicted in Figure 5D, the root activity was significantly reduced in DR-stressed plants by 43.22% in contrast to normal irrigated seedlings. Nonetheless, the supplementation of ME markedly enhanced the root activity by 29.33% compared with only DR-stressed seedlings (Figure 5B). proline and Soluble sugars content were remarkably increased (124.12%, 32.59%, respectively) under the DR group compared with CK plants. Per contra, ME treatment considerably reduced the proline content by 20.09% and 11. 24% compared with DR-stressed seedlings, respectively (Figure 5C,D). 

### 3.5. Oxidative Damage

**Figure 6:**
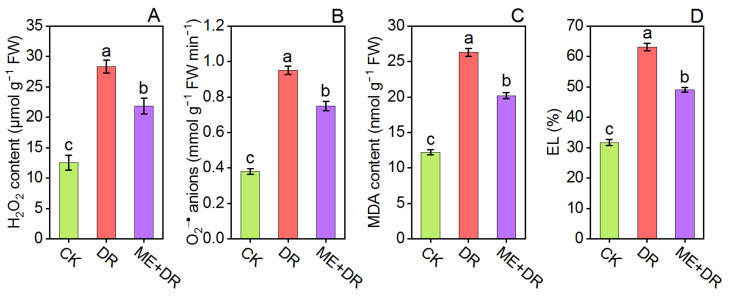
Exogenous supplementation of melatonin reduces oxidative damage biomarkers [H_2_O_2_ content (**A**), O_2_^•−^ anions (**B**), MDA content (**C**), and EL (**D**)] of tomato seedling under drought stress conditions. Means ± standard error, *n* = 3, significant difference is exhibited by lowercase letters (*p* ≤ 0.05), according to LSD test.

After two weeks of DR treatment, the H_2_O_2_, O_2_^•−^, MDA, and EL levels were measured in the leaves of tomato seedlings (Figure 6). For instance, in the tomato plants under the DR environment, the H_2_O_2_, O_2_^•−^, MDA, and EL levels significantly increased by 1.25-, 1.50-, 1.26-, and 0.99-fold, respectively, compared with well-water seedlings. Importantly, ME-pretreated plants subjected to DR-stress reduced this content only by 0.22-, 0.21-, 0.23-, and 0.22-fold, respectively, compared with the DR-stressed group (Figure 6).

### 3.6. Antioxidant Enzymes Activity and Gene Expression

**Figure 7:**
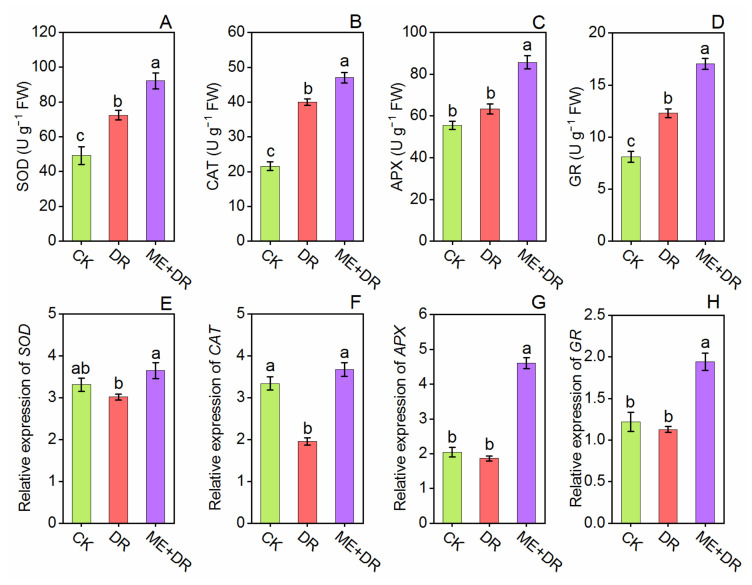
Exogenous supplementation of melatonin promoted antioxidant enzymes [SOD (**A**), CAT (**B**), APX (**C**), GR (**D**)] and their encoding genes [*SOD* (**E**), *CAT* (**F**), *APX* (**G**), *GR* (**H**)] of tomato seedlings under drought stress condition, as shown by leaf’ analysis. Means ± standard error, *n* = 3, significant difference is exhibited by lowercase letters (*p* ≤ 0.05), according to LSD test.

**Figure 8:**
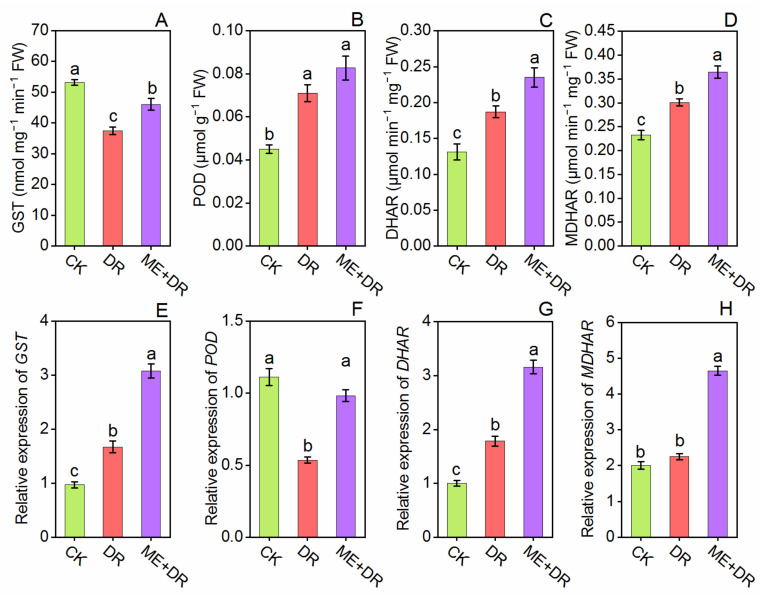
Exogenous supplementation of melatonin promoted antioxidant enzymes [GST (**A**), POD (**B**), DHAR (**C**), MDHAR (**D**)] and their encoding genes [*GST* (**E**), *POD* (**F**), *DHAR* (**G**), *MDHAR* (**H**)] of tomato seedlings under drought stress condition, as shown by leaf’ analysis. Means ± standard error, *n* = 3, significant difference is exhibited by lowercase letters (*p* ≤ 0.05), according to LSD test.

The antioxidant enzymes’ (APX, CAT, GR, GST, POD, SOD, DHAR, and MDHAR) activities were measured in the leaves of tomato plants (Figure 7 and Figure 8). By exposure to DR stress, the SOD, CAT, APX, GR, and POD, enzymes activity was enhanced by 47.15-, 85.39-, 14.03-, 51.54-, and 57.77%, respectively; activity of GST enzyme was decreased by 29.51% compared to CK seedlings. It is noteworthy that when ME-treated seedlings were subjected to DR-treatment, this further elevated these antioxidant enzymes by 27.24-, 17.63-, 35.24-, 38.64-, 40.01-, and 23.02% respectively, compared with the DR-stress group (Figure 7A–D and Figure 8A,B). Moreover, subject to the DR group, the DHAR and MDHAR activity noticeably increased by 42.74- and 29.18%, respectively, compared with well-watered seedlings (Figure 8C,D). Conversely, ME pretreatment along with DR-group, further increased the DHAR and MDHAR activities by 25.66- and 21.26%, respectively, compared with DR-stressed seedlings.

We further measured the transcriptional levels of genes related to antioxidant enzymes (APX, CAT, DHAR, GST, GR, MDHAR, POD, and SOD) (Figure 7E–H and Figure 8E–H). The tomato plants exposed to DR-stress significantly amplified the relative gene expression level of these enzymes. Per contra, ME-treated plants exposed to DR-stress added more increments in the transcriptional levels of these enzyme-related genes, when compared to DR and CK seedlings (Figure 7E–H and Figure 8E–H). These outcomes suggested that exogenous ME application alleviated DR-stress tolerance by enhancing the mRNA pattern of genes of these detoxifying enzymes.

**Figure 9:**
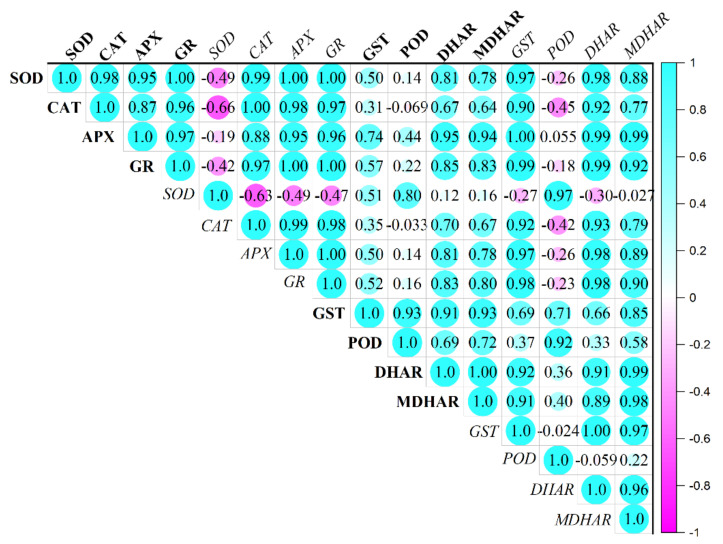
Pearson correlation analysis of antioxidant enzymatic activity (illustrated in bold font) and genes (illustrated in italics font) related to it.

We also performed a Pearson correlation analysis of eight enzymes and their related genes. Our results revealed that the expression of the antioxidant gene was positively correlated with the drought stress condition and its expression was higher when exogenous melatonin was applied to the tomato plant (Figure 9). Similarly, the activity of antioxidant enzymes of the Halliwell Asada pathway was reported to have a positive correlation with the drought condition (Figure 9). Moreover, the expression of genes *viz*., *SOD*, *CAT*, *APX* and *GR* was reported to have a positive correlation (expressed in the blue color shade in Figure 9) with their respective enzymatic activity. However, there was also a negative but not strong correlation when SOD activity was compared with CAT, APX and GR activity. In most of the cases, the expression of eight antioxidant genes was reported to have a positive correlation with the enzymatic activities.

**Figure 10:**
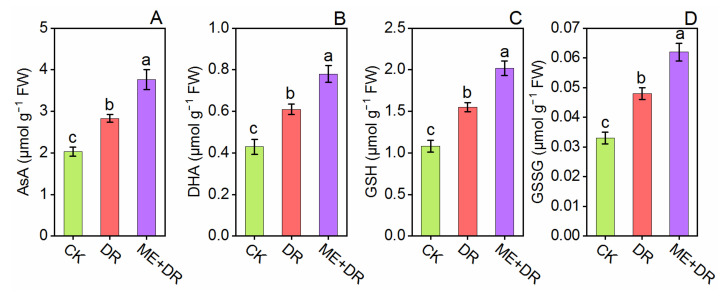
Exogenous supplementation of melatonin promoted non-enzymatic antioxidant [AsA (**A**), DHA (**B**), GSH (**C**), and GSSG (**D**)] system of tomato seedlings under drought stress conditions. Means ± standard error, *n* = 3, significant difference is exhibited by lowercase letters (*p* ≤ 0.05), according to LSD test.

Under DR stress, the metabolites and enzymes of the Haliwell Asada pathway were also reported to be enhanced significantly which affects the enzymatic and non-enzymatic antioxidant mechanisms in the tomato plant. Compared with CK seedlings, DR-treatment markedly increased the AsA, DHA, GSH, and GSSG content in leaves by 37.37%, 40.46, 43.51%, and 45.45%, respectively (Figure 10). Importantly, ME (100 µM) application subjected to the DR-group further increased the AsA, DHA, GSH, and GSSG content in leaves by 33.21%, 30.29%, 30.32%, and 29.16%, respectively, (Figure 10). These results suggested that antioxidant enzymes helped to reduce oxidative damage and increased the DR-stress tolerance.

## 4. Discussion

The current study aimed to elucidate the functions applications of exogenous ME in tomato seedlings under DR stress. Plants could circumvent oxidative stress damages created by DR stress by improving their antioxidant capacity. However, if their antioxidant capacity is weak, and supplementation of some exogenous compounds having high antioxidant properties could help them to increase tolerance level to particular stress condition [18]. Amid such compounds, ME is the one posing antioxidant properties, thus it can increase stress resistance. As mentioned by Arnao and Hernández-Ruiz [45], under abiotic stresses, ME might act as a signal molecule, as it upregulates anti-stress genes and endogenous ME levels under stress conditions. Moreover, recently published reports suggested that ME has shown a protective role against DR stress in *E. japonica* [22], *A. rosea* [21], and *A. deliciosa* [20]. 

The tomato seedlings subjected to DR treatment significantly decreased growth characetristics. Conversely, ME supplementation notably enhanced plant growth attributes (Figure 2). Recent studies suggest that the growth and biomass production were markedly inhibited by heavy metal stress such as Ni stress in tomato seedlings. In contrast, the growth traits were markedly reinforced by ME application [17,46]. Foliar application of ME remarkably accelerated the vegetative growth of tomato seedlings under DR stress [24]. Moreover, exogenous ME ameliorated growth of *Cucumis sativus* [40] and *Trigonella foenum* [7], under DR stress condition. 

During photosynthesis in the plant, carbohydrates are considered as the main supply, except for another substrate. As reported by Takahashi and Murata [47], under a stress environment, the rate of carbohydrates synthesis was reduction. Among the tomato seedlings exposed to DR stress, ME-pretreated seedlings showed an improvement in leaf photosynthesis, compared with those which were not pretreated with ME (Figure 3). Under drought stress, outcomes of the experiment carried out by Sharma et al. [26] on grafted Chinese hickory plants revealed improved photosynthetic efficiency, enhanced growth and successful recovery of chlorophyll content by the application of exogenous ME. The plethora of studies described that exogenous ME treatment recover leaf photosynthesis in *A. rosea*, *A. deliciosa* and *G. max* under DR stress [9,21,25], and *S. lycopersicum* under vanadium toxicity [43,48]. SPAD index and photosynthetic pigments including Chl a, Chl b, and Caro are necessary for the photosynthetic process which is suggested to be reduced significantly under DR stress. Interestingly, ME application robustly improved the content of the pigment of tomato seedlings (Figure 3). These results are supported by the fact that DR stress led to a reduction in leaf photosynthetic pigment, with ME foliar application alleviating these changes, and thus, ME-application might be a promising tool for mitigating DR stress in *A. rosea* [21], *Dracocephalum moldavica* [10], and *C. tinctorius* [23]. Plants obtain more energy due to increased photosynthetic capacity, which enables them to cope with environmental stresses. 

Roots not only provide structural support to the aerial part of plants, but also supply nutrients and water. Thus, a plant’s survival depends on its appropriate growth, development, and root functions. Drought stress significantly reduced the root growth of tomato [28]. In this work, DR treatment negatively affected the root morphological traits by decreasing root surface area, volume, length, root crossings, tips, forks, diameter, and projected area. Conversely, tomato roots pretreated with ME evidently enhanced root characteristics (Figure 4) contributing to better growth of tomato plants. Similarly, Altaf et al. [33] revealed that melatonin application dominantly enhanced the root architecture system of tomato seedling under NaCl stress. Moreover, the positive relationship between the ME application and root growth has been well-known in *S. lycopersicum* [17], *Citrullus lanatus* [49], and *Stevia rebaudiana* [50] under abiotic stresses. Interestingly, when the tomato seedlings were exposed to DR-stress, root activity declined remarkably, showing strict association to the nutrients uptake and water withholding. Per contra, ME pretreated plants repaired the roots from destruction, thus maintaining proper function of roots (Figure 5). Our study is concordant with the previously published reports where it was suggested that abiotic stress-mediated damage to the root was reported to be ameliorated by the application of exogenous ME [43].

Under drought stress, amid various accentuated responses, osmotic regulation is the most important [51]. The reduced leaf water content under DR stress leads to increment of two main osmoprotectants viz. proline and soluble sugars [52] in *S. lycopersicum* [24] and *C. cathayensis* [26]. Our results also affirm this finding (Figure 5). Nevertheless, the application of ME, particularly via root irrigation, declines levels of proline and soluble sugars. Thus, as indicated by our results, a positive turgor pressure and water balance may be maintained by ME. 

Zhang et al. [40] elaborated that on the cell membrane, excess ROS caused peroxidation of pigments and lipids, ultimately upsurging the cell membranes’ permeability and causing functional damages. The ROS (H_2_O_2_ and O_2_^•−^) content, MDA, and EL have been used as oxidative damage biomarkers. In this study, the oxidative damage levels, as determined by H_2_O_2_, O_2_^•−^, EL and MDA, were increased in tomato seedlings subjected to DR stress. Conversely, exogenous ME application effectively protected plant cells from oxidative damage (Figure 6). In a previous study, Sharma et al. [26] and Sadak et al. [27] exhibited significant lowering of MDA content and decreased oxidative stress by ME-pretreatment in *C. cathayensis* and *Moringa oleifera* plants, respectively, under DR stress. Similarly, Gao et al. [53] revealed that exogenous ME application strikingly declined the level of ROS and MDA content in peach fruit. The results of our study exhibited a reduction in oxidative damage by ME application under DR stress, which is also affirmed by extended literature on various plant species such as *Citrullus lanatus* [49], *Cucumis sativus* [54], and *Stevia rebaudiana* [50], under environmental stresses. Thus, it can be concluded that ME applications can lead to reduced oxidative damage and repairing of disrupted cellular membrane induced by salinity by balancing ROS.

Antioxidant enzymes play a principal role in the defense system of plants against biotic and abiotic stress conditions. Mittler [55] described that under different stresses, increased antioxidant enzymes activities lead to potential and specific ROS scavenging. Melatonin is a multi-regulatory molecule and is recognized as a universal antioxidant [56], because it strengthens plants’ antioxidant defense system and enhances tolerance, mainly by detoxifying excess ROS, which is otherwise induced by environmental stresses [57]. Melatonin noticeably improved the activity of antioxidant enzymes (APX, CAT, DHAR, GR, GST, MDHAR, POD, and SOD) and their relative genes expression (Figure 7 and Figure 8). Altaf et al. [58] revealed that in tomato seedlings, ME surprisingly enhanced the antioxidant machinery by reduction of over-accumulation of ROS, which is primarily due to enhanced resilience to nickel toxicity. Similarly, the upregulation in relative gene expression of *APX*, *CAT*, *DHAR*, *GR*, *GST*, *MDHAR*, *POD*, and *SOD* was observed in ME pretreated tomato seedlings under nickel toxicity [43]. Moreover, the literature exhibited significant enhancement of antioxidant enzymes’ activities by ME under abiotic stresses in various plant species [26,47,59]. Furthermore, the production and accumulation balance of ROS is maintained by ME, because the performance of the antioxidative system gets boosted and the activity of antioxidative enzymes gets triggered by ME application [10,60]. 

On the other hand, in plant tissue, AsA and GSH is a well-known antioxidant, proving ME to be a dynamic antioxidant [61]. Wang et al. [60] reported that under environmental stresses, significant changes occur on AsA and GSH content. The present work revealed that ME supplementation predominantly elevated the AsA and GSH content. Furthermore, ME application significantly enhanced the DHA and GSSG content in tomato seedlings (Figure 10). Similarly, ME application sharply enhanced the AsA and GSH contents under Ni toxicity [58], under NaCl stress [62], and under NaHCO_3_ stress [63] in *S. lycopersicum*. Summarizing the discussion, our results revealed that ME alleviated the negative impact of DR stress on tomato seedlings’ growth by improving photosynthesis, root architecture, antioxidant defense system and by regulating the expression of antioxidants-related genes.

## 5. Conclusions

**Figure 11:**
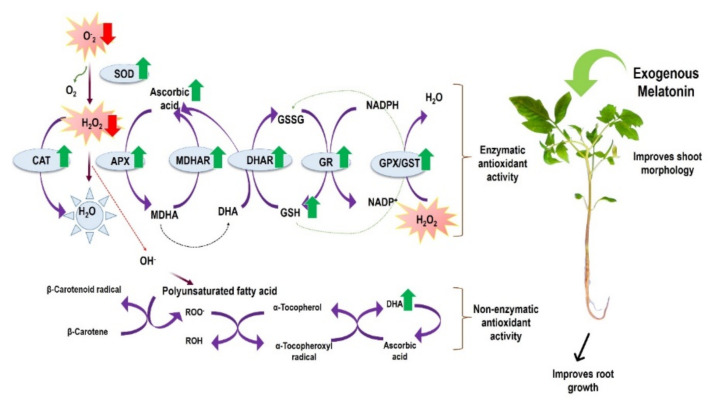
A proposed model showing how melatonin mitigates drought stress tolerance in tomato seedlings.

The present study explored the collective decline in drought stress resilience in plants due to impaired photosynthetic activities, severe growth retardation, excess ROS accumulation, and damaged root morphology under drought stress (Figure 11). Per contra, the stress induced by drought was effectively mitigated by exogenous application of melatonin, as oxidative damage is reduced (with refining in antioxidant defense system and inhibiting ROS production), root architecture is enhanced, photosynthesis efficiency is strengthened and thus growth attributes are recovered. In addition, the soluble sugars and proline content noticeably decline, and the formation of AsA-DHA and GSH-GSSG arere prominently improved in ME-pretreated tomato plants. This suggests that exogenous ME is an effective protectant that improves DR tolerance in tomato seedlings by enhancing antioxidant enzymes and reducing oxidative damages. Nevertheless, under drought stress and melatonin-mediated resilience in plants, and its mechanism should be investigated in-depth, along with the explicit study of molecular approaches.

## Data Availability

Not applicable.

## Supplementary Materials

The following are available online at https://www.mdpi.com/article/10.3390/antiox11020309/s1, Table S1: The detail of primers.
