# Supplementary material for: Melatonin Improves Drought Stress Tolerance of Tomato by Modulating Plant Growth, Root Architecture, Photosynthesis, and Antioxidant Defense System
Source: Antioxidants (Basel). 2022 Feb 3;11(2):309. doi: 10.3390/antiox11020309 (PMC8868175; doi:10.3390/antiox11020309)
Supplement: Supplementary file 1 [file antioxidants-11-00309-s001.zip › antioxidants-1556158-supplementary.pdf]

Table S1. The detail of primers.

Gene acronym	Accession number	Forward primer	Reverse primer
actin	Solyc03g078400	TGGTCGGAATGGGACAGAAG	CTCAGTCAGGAGAACAGGGT
SOD	Solyc02g082590	ACTACTCCCAGTTGCATCCC	CACCAGGAGCAGCCATGATA
CAT	Solyc12g094620	GCAGCTCCCAGTTAATGCTC	AGCAGGACGACAAGGATCAA
APX	Solyc06g005150	GGCACTCTGCTGGTACCTAT	GGAGAGAGTGGGAAACTGCT
GR	Solyc09g065900	GGAGCCATAGAGGTTGACGA	CTCCTCCCTCCATCAAAGCA
MDHAR	Solyc08g081530	CGGACAGTTCCGAACAAACA	CCCGTGCAATTCGGTTGTAT
DHAR	Solyc05g054760	GAGGTGAACCCTGAAGGGAA	CCCACAGAGGCAAATTCAGG
GST	Solyc05g006740	TTGTCTGGTGCTCTCTTCGT	TGAGAGCAACTGGAGCATGA
POD	Solyc07g052510	GGTCTGTTCCAATCCGATGC	CACCAGCACTCCCTGTCTTA
